# Potential Drug-drug Interactions in Acute Ischemic Stroke Patients at the Neurological Intensive Care Unit

**DOI:** 10.1515/med-2019-0093

**Published:** 2019-11-07

**Authors:** Dejan Z. Aleksic, Slobodan M. Jankovic, Milos N. Mlosavljevic, Gordana L. Toncev, Svetlana D. Miletic Drakulic, Srdjan M. Stefanovic

**Affiliations:** 1 University of Kragujevac, Serbia, Faculty of Medical Sciences, Kragujevac, Serbia; 2 University of Kragujevac, Serbia, Faculty of Medical Sciences, Department of Pharmacology and toxicology, Kragujevac, Serbia; 3 University of Kragujevac, Serbia, Faculty of Medical Sciences, Department of Neurology, Kragujevac, Serbia; 4 University of Kragujevac, Serbia, Faculty of Medical Sciences, Department of Pharmacy, Kragujevac, Serbia

**Keywords:** Online checkers, Risk factors, Risperidone

## Abstract

**Background:**

Clinically relevant potential drug-drug interactions are considered preventable adverse drug reactions.

**Objective:**

The aim of this study was to ascertain the frequency of potential drug-drug interactions in acute ischemic stroke patients and to explore factors associated with occurrence of potentially contraindicated drug-drug interactions.

**Methods:**

This observational retrospective cohort and nested case-control study was carried out among patients treated for acute ischemic stroke at the Neurological Intensive Care Unit in the Clinical Centre Kragujevac, Serbia. The potentially drug-drug interactions for each day of hospitalization were identifi ed using Micromedex® soft ware. Based on the existence or absence of potentially contraindicated drug-drug interactions, the participants were divided into a group of cases (n=111) and the control group (n=444).

**Results:**

A total of 696 patients were analysed. All patients had a minimum of one potential drug-drug interaction during hospitalization. The most common drugs involved in potential drug-drug interactions were aspirin (8.02%), diclofenac (7.49%) and warfarin (7.14%). The number of medications prescribed for simultaneous use during hospitalisation and the use of antipsychotics in therapy signifi cantly increased the likelihood of potentially contraindicated drug-drug interactions aft er adjustment by means of logistic regression for 1.2 and 3 times, respectively.

**Conclusions:**

This study suggests that patients with acute ischemic stroke are frequently exposed to potential drug-drug interactions. It is essential to identify potentially drug-drug interactions in these patients as early as possible in order to prevent adverse drug reactions and ensure safe recovery. Besides, full attention should be paid when adding each new medication in therapy, particularly when a neurologist decides to prescribe antipsychotics, such as risperidone.

## 1 Introduction

Potential drug-drug interactions (pDDIs) occur when the effects of one drug may be altered by the effects of another drug(s) during their concomitant use. In a small percentage of cases, drug-drug interactions have a clinical relevance and can lead to serious harm; if recognized in time, they could be avoided by therapy modifications, and that is why they are considered to be preventable adverse drug reactions (ADRs) [1]. Therefore, it is essential to recognize the importance of pDDIs, although in most cases they do not require any action other than additional monitoring [2]. The pDDIs can be classified by severity, depending on the theoretical source of information for their analysis. There are several online checkers varying in regard to sensitivity and specificity to detect pDDIs in routine practice, like Micromedex^®^, Drug Interaction Facts^®^, Lexi-Interact^®^, Pharmavista^®^, EpocratesRx^®^, etc [3]. These pieces of software are very practical and applicable in everyday clinical work as they not only reveal the pairs of medications involved in pDDIs, but also classify pDDIs according to severity, e.g. the Micromedex® 2.0 Drug-Reax System differentiates between potentially contraindicated drug-drug interactions (pCDDI), major, moderate and minor pDDIs [4]. It is quite clear that the identification of pCDDI deserves special attention when prescribing drug combinations.

Exposure to contraindicated drug-drug reactions (CDDIs) significantly increases during hospitalisation [5]. This can be explained by advanced age and multimorbidity in the majority of inpatients, as well as by frequently impaired renal and/or liver function, resulting in electrolyte disturbances. In addition, these patients are commonly prescribed many drugs simultaneously, and the main route of drug administration is intravenous [6].

A previous study of pDDIs in 146 patients with acute ischemic and hemorrhagic stroke at admission showed 582 different pDDIs, with a pDDI prevalence of 61% [7]. Additionally, in a study carried out on 200 patients, of whom 190 were treated for ischemic stroke, the prevalence of pDDIs was 89.5% [8]. Multimorbidity and polypharmacy were significantly more common in patients with stroke [9], making these patients very vulnerable regarding the occurrence of pDDIs.

To our best knowledge there are no published studies about risk factors for pDDIs/pCDDIs among stroke patients during their hospitalization. The studies on hospitalized patients with other diagnoses showed that the total number of prescribed drugs [1,2,6,10], the length of hospitalisation [1,6] and older age [6] were the most frequently associated with pDDIs.

The objective of our study was to determine the frequency of pDDIs in acute ischemic stroke patients and to identify the type of drugs involved in the most common pDDIs, as well as to explore factors associated with the pCDDIs.

## 2 Materials and methods

This observational retrospective cohort and nested case-control study was conducted among consecutively enrolled inpatients with ischemic stroke treated at the Neurological Intensive Care Unit (NICU) of the Clinical Center of Kragujevac (CCK), Serbia, during the period from 01/01/2012 to 31/12/2014. The study was approved by Ethics Committee of the CCK (No. 01/8745).

The data were collected from the patient files. The inclusion criteria were: adult patients (older than 18 years) who had been treated in the NICU, those with a diagnosis of acute ischemic stroke (ICD-10 code I63.0-I63.5), and those who were prescribed two or more drugs during hospitalization. The following patients were excluded from the study: patients who were treated in the NICU for less than 7 days, those who had some other primary illness (i.e. status epilepticus, malignant disease, inflammatory CNS disorders, multiple sclerosis, Guillain-Barré Syndrome, etc.), and those with incomplete data in their medical files.

The pDDIs for each day of hospitalization at the NICU were identified by means of Micromedex^®^ software [4]. A systematic review showed that Micromedex® was the most commonly used drug-drug interaction software primarily due to its high sensitivity (≥83%) and specificity (≥90%) [3]. According to this online checker, pDDIs were categorized by severity (i.e. contraindicated, major, moderate, and minor) and by scientific evidence (as excellent, good, and fairly proved).

Contraindicated pDDIs with excellent or good literature evidence support has the most relevance for clinical practice as such drug combinations would inevitably lead to treatment failure or adverse drug events imposing the greatest risk of morbidity and mortality. Slightly less significant pDDIs are contraindicated interactions which were fairly proven based on literature data and major pDDIs which could be life-threatening and/or seek for an efficacious intervention to prevent serious adverse effects. The occurrence of moderate pDDIs increases the risk of exacerbation of patient’s condition to a certain extent and usually requires changes of pharmacotherapy. Minor pDDIs are of the least clinical significance and usually do not require changes of therapy. The total number of pDDIs, number of pDDIs per patient, and frequency of individual drug pairs involved in pDDIs were determined, followed by the most common drug pairs within all pDDI categories. When a particular drug was not available in the Micromedex^®^ database (21 drugs) its pDDIs were not taken into account. All study investigators extracted data from patients’ records, while two clinical pharmacologists, i.e. SJ and SS, independently reviewed and completely agreed with the classification of pDDIs according to evidence and severity.

In accordance to the specific outcome of interest, i.e. the existence or absence of pCDDI, the patients enrolled in the retrospective cohort were divided into two groups: cases where the participants had at least one pCDDI, while the control group consisted of those without exposure to pCDDI, and who were individually matched to each patient in group of cases by gender and age (± 3 years). For each case from the study cohort we first matched by sex and age (±10 years) all available potential controls from the same cohort. Actual controls used in the study (four for each case) were then chosen randomly, step by step for each case. Cases and controls were then explored in terms of differences in previous exposure to putative risk factors for the occurrence of pCDDI.

The following factors were examined for their contribution to pCDDIs in NICU: sex (male; female); age (in years); transfer from another department to NICU; indication for hospital admission; diagnosis at discharge; number of diagnoses assigned to a patient during hospitalization; the influence of existing co-morbidities (such as diabetes mellitus, liver disease, malignancy, AIDS, chronic kidney disease, chronic congestive heart failure, myocardial infarction, hemiplegia, cerebrovascular disease, peripheral vascular disease, dementia, chronic obstructive pulmonary disease, connective tissue disease and peptic ulcer disease) on a 10-year risk of mortality, which was assessed using the Charlson Comorbidity Index (CCI) with and without adjustment for age [11]; occurrence of recurrent stroke; length of hospitalization (hospitalization only in NICU, in days); body temperature (at least one episode of body temperature above 38ºC during NICU stay was considered to be fever); glycaemia (all values measured during hospitalization were used and the average value per patient during hospitalization was calculated) and disturbances of glucose level during the stay in NICU (an average value of glycaemia above 6.1 mmol/ L was considered as abnormal); the estimated glomerular filtration rate (GFR) was calculated using the Modification of Diet in Renal Disease-MDRD) equation [12]. Acute kidney injury during stroke was defined as GFR below 60 ml/min/1.73 m^2^ during the stay in the NICU; number of prescribed drugs and polypharmacy (five or more medications daily). All prescribed medications during the hospitalization in the NICU were classified according to the Anatomical Therapeutic Chemical (ATC) classification and the therapeutic group of drugs); certain groups of drugs used for acute ischemic stroke and/ or its complications (dual anti-platelet therapy, anticoagulant therapy (divided into separate groups based on whether the patients were switched to oral anticoagulants), statins, antidepressants, antip-sychotics, anticonvulsants, antibiotics); data indicating the severity and scientific evidence of pDDIs other than pCDDI (i.e. major, moderate, minor, excellent, good and fair evidence).

An a priori calculation of the required sample size for this nested case-control study was performed using a Z test for assessing the differences between two independent proportions and the following baseline parameters: i) the power of the study of at least 80%; ii) the probability of an error type I of 5% (α=0.05); and iii) the effect size, taken from the study by Lima et al [13], where more than 9 prescribed drugs per patients was identified as the most important factor associated with the occurrence of pDDI. At that, 64% patients in the group of cases and 48% of controls used > 9 drugs simultaneously. Using the commercial software package G*Power [14] a total sample size of at least 370 participants was obtained, if the distribution between compared groups had been 4:1 in favor of controls (i.e. 74 cases and 296 controls).

The IBM Statistical Package for Social Sciences (SPSS Inc, Chicago IL), version 20, was used to perform all statistical analyses. The continuous data were summarized as means with standard deviations (mean ± SD) or medians, interquartile ranges (IQRs) and minimum and maximum values depending on the normality of data distribution, which had been tested by the Kolmogorov-Smirnov test. The categorical variables were presented as frequencies and percentages. Student’s T-test and the Mann-Whitney U test were used for examining the differences between continuous variables. The Chi-square test (χ^2^) was used for comparisons of categorical variables. The Spearman´s correlation coefficient was used for determining the direction and strength of the connection between certain continuous variables and the total number of pDDI. The influence of confounding and independent variables on the occurrence of dichotomous outcome (i.e. pCDDI) was calculated using a conditional logistic regression model with a backward stepwise selection of predictors. The strength of an association is expressed by crude and adjusted odds ratio (OR) with 95% confidence interval (95% CI). Significant association was assumed if 95% CI had not included the value of 1. The p <0.05 was considered a statistically significant value for all analyses.

## 3 Results

**Figure 1:**
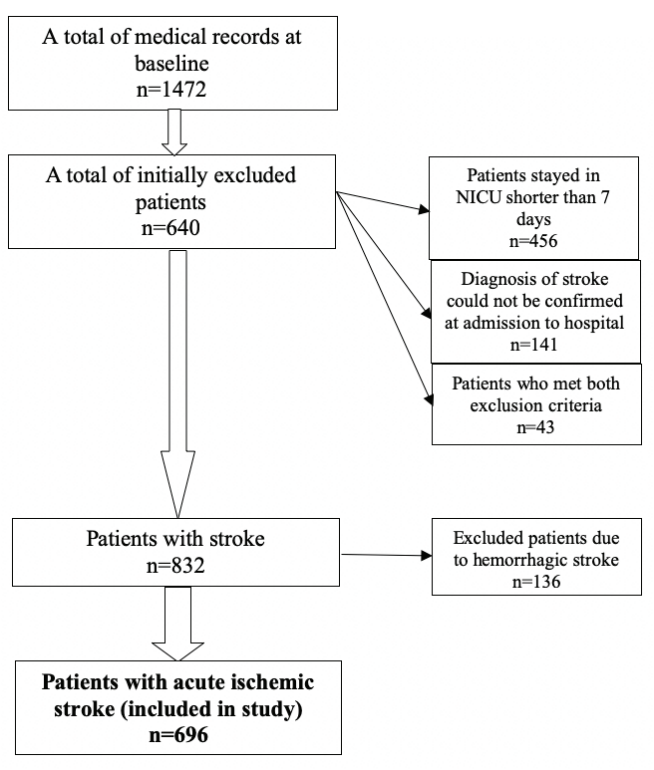
Patients´ selection process.

A total of 1472 consecutively chosen medical records of patients treated in the NICU were analyzed. Six hundred and forty (43, 48%) medical records were excluded from the study: 456 (30%) patients due to stay in the NICU for less than 7 days, 141 (9.58%) patients because the diagnosis of stroke could not be confirmed at admission to hospital and 43 (2.92%) patients who met both exclusion criteria. The remaining 832 (56.52%) patients had a stroke: 136 (9.24%) of them were with hemorrhagic and 696 (47.28%) with acute ischemic stroke (Figure 1).

In the sample of patients with ischemic stroke, there were more women (53.4%), with a median age of 77.00

(IQR=70-82) and 85.6% of patients were aged ≥65 years. These and other demographics, clinical characteristics of patients and characteristics of hospitalization are shown in Table 1. Over the observation period, 224 different drugs were administered to all patients during their stay in the NICU.

**Table 1:** Characteristics of cohort with acute ischemic stroke.

VARIABLES	VALUES n (%) of median (IQR^1^)
**Demographic characteristics**	
Number of patients	696 (100)
Age (years)	77 (70-82)
Sex (M/F^2^)	324/372 (46.6/53.4)
**Characteristics of hospitalization**	
Transfer from another ward	2 (0.28)
Length of hospitalization in NICU^3^ (days)	Median 14.00
	IQR: 10-19
Death	248 (35.6)
**Comorbidities**	
Number of diagnosis	Median 5.00
	IQR: 4-6
Fever	213 (30.6)
Recurrent stroke	84 (12.1)
Hypertension	530 (76.1)
Cardiomyopathy	283 (40.7)
Atrial fibrillation	230 (33.0)
Diabetes mellitus type 2	205 (29.5)
Disturbances of glucose level during hospitalization in NICUwithout DM2^4^	225 (45.9)
Chronic kidney disease	124 (17.8)
Acute kidney injury during hospitalization in NICU	253 (44.7)
Glomerulal filtration rate (ml/min/1.72m^2^)	Median 57.00
	IQR: 35.75-77
Liver chirosis	2 (0.28)
Pneumonia	66 (9.5)
Chronic obstructive pulmonary disease	34 (4.9)
Delirium	65 (9.3)
Epilepsy	65 (9.3)
Coma	31 (4.5)
Dementia	26 (3.7)
Anemia	37 (5.3)
Age-adjusted Charlson Comorbiditiy Index (with influence of ages)	Median 6.00
	IQR: 5-8.
Charlson Comorbiditiy Index (without adjustment of age)	Median 3.00
	IQR: 2-5.
**Number and type of prescribed medications during hospitalization in NICU**	
Number of drugs prescribed	Median 17,00
	IQR: 14-20
Number of pharmacological/therapeutic subgroups of drugs prescribed according to ATC^5^ classification	Median 6.00
	IQR: 5-6
Polypharmacy	
5-8 drugs	10 (1.4%)
≥9 drugs	686 (98.6%)
Anticoagulant therapy	422 (60.7)
Anticoagulant therapy without OAC^6^	360 (51.8)
Anticoagulant therapy with OAC	51 (7.3)
Dual antiplatelet therapy	148 (21.3)
Antibiotics	552 (79.3)
Antipsychotics	141 (20.3)
Antidepressants	29 (4.2)
HMG-CoA^7^ reductase inhibitors (statins)	149 (21.4)

1 - interquartile rang ^2^- male/female ^3^- Neurological Intensive Care Unit ^4^- diabetes mellitus type 2 ^5^- Anatomical Therapeutic Chemical ^6^- oral anticoagulant ^7^**-** hydroxymethylglutaryl-coenzyme A

The average number of pDDI was 14.84 ± 9.01 (minimum 1, maximum 66). The total of 10368 pDDIs was observed in entire sample of patients including all days of hospitalization in NICU, of which there were 561 different combinations of medications. Characteristics of pDDIs of the cohort with acute ischemic stroke are shown in Table 2. A significant correlation between the total number of pDDIs and the length of hospitalization in NICU was observed, (p<0.001, r=0.305). However, the relationship between the total number of pDDIs and the number of drugs used concomitantly was also significant and positive (p<0.001, r=0.779).

**Table 2:** Characteristics of potential drug-drug interactions pDDI1 of cohort with acute ischemic stroke.

Variables	n (%) of patients/median (IQR^2^)
pDDI	Median 13.00
	IQR: 9-19
Contraindicated pDDI	111 (15.95)
Major pDDI	680 (97.70)
Moderate pDDI	671 (96.41)
Minor pDDI	571 (82.04)
Excellent evidence of pDDI	643 (92.38)
Good evidence of pDDI	669 (96.12)
Fair evidence of pDDI	680 (97.70)

pDDI^1^-potential drug-drug interaction IQR^2^-interquartile range

The cardiovascular drugs (C category according to ATC classification) were found to have the highest prevalence among all medications that are involved in observed pDDI (197different pDDI, i.e. 35.12% of total pDDI).

Twelve different drugs were responsible for 362 different pDDIs (65.57%). The most common drugs involved in pDDIs were aspirin (45 pDDIs, 8.02%), diclofenac (42 pDDIs, 7.49%) and warfarin (39 pDDIs, 7.14%). Dual antiplatelet therapy (aspirin-clopidogrel) was recorded in 21.26% of patients. Risperidone was the most commonly used antipsychotic (19.83%) and it was potentially involved in interactions mostly with ranitidine (115 patients, 16.58% of patients) and with metoclopramide (which was identified as contraindicated pDDI in 2.30% of patients). The selective serotonin reuptake inhibitors (SSRIs) were the most commonly used antidepressants (3.5%). pDDIs between SSRIs and aspirin and other non-steroidal anti-inflammatory drugs (NSAIDs) were recorded in 2.4% and 4.74% of patients, respectively.

At least one pCDDI occurred in 111 patients (15.95%). Ninety-nine (15.66%) patients had one pCDDI, 11 (1.58%) patients had two pCDDIs and one (0.14%) patient had three pCDDIs.

Table 3 shows the most common pDDI pairs according to severity of pDDI. The most frequent pCDDIs were ceftriaxone-Ringer´s solution (calcium gluconate) (8.62%), ketorolac-aspirin (3.88%) and metoclopramide-risperidone (2.30%), while a combination between aspirin and diclofenac (55.32%), diclofenac and furosemide (38.79%) and aspirin and furosemide (40.66%), were identified as the most common major pDDI.

**Table 3:** All contraindicated potential drug-drug interactions pDDIs and the most frequent major, moderate and minor pDDIs.

No.	Drug 1	Drug 2	Scientific Documentation (evidence)	No (%) of patients
**Contraindicated**	**pDDI**			
1	Ceftriaxone	Ringer´s solution	Good	47 (6,75)
2	Ketorolac	Aspirin	Fair	27 (3,88)
3	Metoclopramide	Risperidone	Fair	16 (2,30)
4	Ceftriaxone	Calcium gluconate	Good	13 (1,87)
5	Ketorolac	Diclofenac	Fair	6 (0,86)
6	Ringer´solution	Atropine	Fair	3 (0,43)
7	Clarithromycin	Simvastatin	Good	2 (0,28)
8	Fluconazole	Amiodarone	Fair	2 (0,28)
9	Potassium chloride	Atropine	Fair	2 (0,28)
10	Potassium chloride	Amantadine	Fair	1 (0,14)
11	Ketorolac	Ibuprofen	Fair	1 (0,14)
12	Ketorolac	Pentoxifylline	Fair	1 (0,14)
13	Metoclopramide	Chlorpromazine	Fair	1 (0,14)
14	Metoclopramide	Fluoxetine	Fair	1 (0,14)
15	Metoclopramide	Trazodone	Fair	1 (0,14)
**Major pDDI**				
1	Aspirin	Diclofenac	Fair	385 (55,32)
2	Diclofenac	Furosemide	Good	270 (38,79)
3	Aspirin	Furosemide	Good	283 (40,66)
4	Diclofenac	Enoxaparine	Good	194 (28,16)
5	Clopidogrel	Diclofenac	Fair	150 (21,55)
**Moderate**	**pDDI**			
1	Aspirin	Bisoprolol	Good	242 (34,77)
2	Bisoprolol	Diclofenac	Good	199 (28,29)
3	Aspirin	Ramipril	Fair	159 (22,84)
4	Diclofenac	Enalapril	Excellent	121 (17,38)
5	Diclofenac	Ramipril	Excellent	136 (19,50)
**Minor pDDI**				
1	Aspirin	Ranitidine	Excellent	499 (71,69)
2	Aminophylline	Ranitidine	Good	189 (27,15)
3	Aminophylline	Furosemide	Fair	158 (22,70)

A fatal outcome was more common in the group experiencing pCDDIs than in the group without exposure to such an event (43.2% vs. 32.4%, χ^2^=4.121 p=0.022).

The differences between cases (n=111) and controls (n=444) in terms of their baseline demographic and clinical features, as well as types of medication therapy and characteristics of pDDI (except pCDDI), are shown in Tables 4.a, 4.b The prevalence of most risk factors and confounding variables did not differ statistically significantly between these two groups of patients. The higher percentage of patients with fever during hospitalization in the NICU and patients with disturbances of glucose levels during their stay in the NICU in the group of cases, was observed (Table 4.a) Furthermore, the greater number of diagnoses, longer hospitalization in the NICU and a higher number of prescribed drugs were observed in the group of cases compared to the controls (Table 4.a) Finally, dual antiplatelet therapy, antibiotics and antipsychotics were a more commonly prescribed medication in the group of cases (Table 4.b)

**Table 4A:** Baseline demographic and clinical characteristics of cases and controls.

Variables	Cases n=111 (25%)	Controls n=444 (75%)	Test and value p	Crude OR^1^ with 95% CI^2^
Demographic characteristics				
Sex |Male Female	***54 (48.6%)***	216 (48.6%)	χ23=0.000	OR=1.000
	***57 (51.4%)***	228 (51.4%)	p=1.000	(0.660-1.516)
	Median 74.00	Median 76.00	U^5^=22750	
**Age (years)**	(IQR^4^=68-81)	(IQR=71;81	Z=-1.253	OR=0.980 (0.958-1.004)
			p=0.210	
	95 (85.6%)	389 (87.6%)	χ^2^=0.171	OR=0.839
**Older than 65 years**			p=0.567	(0.461-1.530)
Comorbidities				
	Median 5.00	Median 5.00	U=18333.000	OR=1.304
**Number of diagnosis**	IQR 4-7	IQR 4-6	Z=-4.251	(1.150 -1.479)
			p=0.000*^6^	
			χ^2^=0.299	OR=1.243
**Recurrent stroke**	16 (14.4%)	53 (11.9%)	p=0.479	(0.680-2.269)
			χ^2^=2.362	OR=1.575
**Hypertension**	92 (82.9%)	335 (75.5)	p=0.096	(0.919-2.701)
**Cardiomyopathy**	42 (37.8%)	172 (38.7%)	χ^2^=0.004	OR=0.963
			p=0.862	(0.627-1.477)
Atrial fibrillation	32 (28.8%)	152 (34.2%)	χ^2^=0.940	OR=0.778
			p=0.279	(0.494-1.227)
**Diabetes mellitus type 2**	43 (38.7%)	130 (29.3%)	χ^2^=3.276	OR=1.527
			p=0.054	(0.990 -2.355)
Chronic kidney disease			χ^2^=2.496	OR=1.555
	26 (23.4%)	73 (16.4%)	p=0.086	(0.937-2.578)
**Chronic obstructive pulmonary disease**	4 (3.6%)	23 (5.2%)	χ^2^=0.197	OR=0.684
			p=0.490	(0.232-2.021)
**Epilepsy**	15 (13.5%)	36 (8.1%)	χ^2^=2.495	OR=1.771
			p=0.078	0.932-3.365)
			χ^2^=0.000	OR=1.055
**Dementia**	5 (4.5%)	19 (4.3%)	p=0.917	(0.385-2.891)
			χ^2^=1.376	OR=1.777
**Anemia**	9 (8.1%)	21 (4.7%)	p=0.159	(0.790-3.996)
	Median 4.00	Median 3.00	U=22382.500	OR=1.093
**Charlson comorbiditiy index without adjustment of age**			Z=-1.518	
	IQR 2-5	IQR 2-4	p=0.129	(0.975-1.226)
***Complications during stay in NI***^7^				
Fever	45 (40.5%)	127 (28.6%)	χ^2^=5.371	OR=1.702
			p=0.015*	(1.106-2.619)
**Disturbances of glucose level without DM2^8^**			χ^2^=4.916	OR=1.879
	39 (57.4%)	131 (41.7%)	p=0.019*	(1.105-3.139)
**Acute kidney injury without CKD^9^**			χ^2^=2.518	OR=1.515
	45 (54.2%)	161 (43.9%)	p=0.087	(0.939-2.445)
**Pneumonia**	13 (11.7%)	39 (8.8%)	χ^2^=0.585	OR=1.378
			p=0.344	(0.708-2.680)
**Delirium**	11 (9.9%)	42 (9.5%)	χ^2^=0.000	OR=1.053
			p=0.885	(0.523-2.118)
			χ^2^=1.984	OR=2.078
**Coma**	8 (7.2%)	16 (3.6%)	p=0.095	(0.866-4.987)
Characteristics of hospitalization and treatment in NICU				
			U=17539.500	
**Length of hospitalization**	Median 17.00 IQR 12-24	Median 13.00 IQR 10-18	Z=-4.708	OR=1.056 (1.031 -1.081)
			p<0.001*	
**Number of prescribed drugs**	Median 21.00	Median 16.00	U=11573.000	OR=1.184
	IQR 18-25	IQR 13-19	Z=p<0.001-8.664	* (1.136 -1.233)

1 - odds ratio ^2^ - confidence interval ^3^ - chi squared test ^4^ - interquartile rang ^5^ - Mann-Whitney U test ^6-^ statistically significant ^7^ - Neurological Intensive Care Unit ^8^- diabetes mellitus type 2 ^9^ - chronic kidney disease

**Table 4B:** Type of therapy and potential drug-drug interactions (pDDI) characteristics of cases and controls.

Variables	Cases n=111 (25%)	Controls n=444 (75%)	Test and value p	Crude OR^1^ with 95% CI^2^
HMG-CoA3 reductase inhibitors (statins)	23 (20.7%)	99 (22.3%)	χ^2 4^=0.053	OR=0.911
			p=0.720	(0.547-1.518)
Anticoagulant therapy	68 (61.3%)	262 (59%)	χ^2^=0.105	OR=1.099
			p=0.666	(0.717-1.682)
			χ^2^=5.285	OR=0.568
Dual antiplatelet therapy	35 (31.5%)	92 (20.7%)	p=0.015*^5^	(0.358-0.900)
Antibiotics	105 (94.6%)	338 (76.1%)	χ^2^=17.674	OR=5.488
			p<0.001*	(2.343-12.855)
Antipsychotics	38 (34.2%)	86 (19.4%)	χ^2^=10.468	OR=2.167
			p=0.001*	(1.372 -3.423)
			χ^2^=0.589	OR=1.593
Antidepressants	7 (6.3%)	18 (4.1%)	p=0.306	(0.648-3.914)
Major pDDI	110 (99.1%)	432 (97.3%)	χ^2^=0.596	OR=3.056
			p=0.262	(0.393 -23.752)
			χ^2^=1.802	OR=4.648
Moderate pDDI	110 (99.1%)	426 (95.9%)	p=0.102	(0.614-35.197)
			χ^2^=0.007	OR=1.067
Minor pDDI	93 (83.8%)	368 (82.9%)	p=0.821	(0.608-1.872)
Excellent evidence of pDDI	104 (93.7%)	411 (92.6%)	χ^2^=0.042	OR=1.193
			p=0.682	(0.513-2.773)
Good evidence of pDDI	110 (99.1%)	423 (95.3%)	χ^2^=2.488	OR=5.461
			p=0.064	(0.727-41.044)
Fair evidence of pDDI	110 (99.1%)	426 (98.2%)	χ^2^=0.064	OR=2.018
			p=0.501	(0.250 -16.308)

1 - odds ratio ^2^- confidence interval ^3^- hydroxymethylglutaryl-coenzyme A ^4^ - chi squared test 5 –statistically significant

The strength of multivariate logistic regression was acceptable (Cox & Snell R square 0.159, Nagelkerke R square 0.261, Hosmer-Lemeshow Chi square 6.436, df=8, p=0.599, overall model accuracy of 82.2%). Although the crude ORs for these factors in univariate regression models had indicated a significant positive relationship with pCDDIs, only the number of medications prescribed for simultaneous use during stay in NICU and the use of antipsychotics in therapy significantly increased the likelihood of pCDDIs after adjustment by means of logistic regression (Table 5), for 1.2 and 3 times, respectively.

**Table 5:** Significant risk factors for potentially contraindicated drug-drug interactions (pCDDI) in stroke patients.

Variables	Crude OR^1^ with 95% CI^2^	Adjusted OR with 95% CI
	OR=1.702	OR=0.942
Fever	(1.106-2.619)	(0.776-1.143)
	p=0.016*	p=0.544
Disturbances of glucose level during hospitalization in NICU3	OR=1.879	OR=0.956
without DM2^4^	(1.105-3.139)	(0.491-1.862-)
	p=0.020*	p=0.895
	OR=0.568	OR=0.600
Dual antiplatelet therapy	(0.358-0.900)	(0.308-1.172)
	p=0.016*	p=0.135
	OR=5.488	OR=1.403
Antibiotics	(2.343-12.855)	(0.484-4.068)
	p<0.001*^5^	p=0.532
	OR=2.167	OR=3.010
Antipsychotics	(1.372 -3.423)	(1.587-5.711)
	p<0.001*	p<0.001*
	OR=1.093	OR=0.940
Charlson comorbiditiy index without adjustment of age	(0.975-1.226)	(0.776-1.139)
	p=0.128	p=0.528
	OR=1.056	OR=0.984 (0.946-1.023)
Length of hospitalization in NICU	(1.031 -1.081)	
	p<0.001*	p=0.408
	OR=1.184	OR=1.202
Number of prescribed drugs	(1.136 -1.233)	(1.120-1.290)
	p<0.001*	p<0.001*

1 - odds ratio ^2^- confidence interval ^3^ - Neurological Intesive Care Unit ^4^- diabetes mellitus type 2 ^5^- statistically significant

## 4 Discussion

The most relevant finding of this research was the very high prevalence of observed pDDIs in patients treated in the NICU, who were exposed to at least one pDDI regardless of severity. Cardiovascular drugs and NSAIDs were the most commonly involved medications in the pDDI. Besides this, approximately one-sixth of patients were prescribed drugs whose simultaneous use was potentially contraindicated, and the presence of such combinations were significantly higher in the deceased. The main identified factors that contributed to exposure to pCDDIs were the number of prescribed drugs and prescription of antip-sychotics.

The reasons for the appearance of pDDIs/pCDDIs in severe patients with acute ischemic stroke relies on potentially inappropriate prescribing due to their exclusively prolonged hospital treatment mostly with the parenteral application of drugs, their functional inability as well as usually identified cardiovascular or other important comorbidities and advanced age of patients. As a rule, such a medical condition often requires the simultaneous use of many drugs which increases the risk of drug interactions. These multiple prescriptions commonly include medications with variable pharmacokinetic features and a narrow therapeutic index, exposing the patients to pCCDIs with its well-known negative consequences, including death.

Other studies that mostly had used Micromedex^®^ as an online drug interaction checker, demonstrated various prevalences of pDDIs in the ICU, ranging from 46.3% to 90.3% [1].However, there was a small number of studies examining their frequency in neurological patients. A study of only 79 such patients showed that the frequency of pDDIs at discharge from the general neurological department was 72% [15], while in patients with stroke (ischemic and hemorrhagic), the prevalence of pDDIs was 61%-89.5% [7,8]. The highest frequency of pDDIs (98% for highly significant interactions), was observed in patients from the ICU among antimicrobial drugs [16], and 95.9% in patients with CKD [17]. A review about the prevalence of pDDIs showed that their frequency was 67% in the ICU and 33% in general departments [1].

A study of pDDIs in stroke patients revealed serious DDIs, potentially associated with the risk of ischemic and hemorrhagic stroke in 17% of patients before hospitalization [7].A number of other studies included seriously ill patients with various other diagnoses who stayed in hospital longer than 24 hours [18] or 48 hours [19], and the likelihood of exposure to pDDI was lower than in our sample. Our sample included patients who stayed in hospital for at least 7 days and since a positive correlation was shown between the length of stay and pDDIs [6,20] the higher frequency of pDDIs in our patients was expected. We noticed exposure to pDDI during the entire hospitalization, not just at admission [15,21] or at discharge, [7,21] which is in line with results of previous studies [19,22]. Also, in certain studies, the frequency of pDDIs was determined only during a specific day of hospitalisation [23] or only at one prescription event per patient [24], so that could be the reason why the frequency of pDDIs in these studies was lower. In addition, relatively large and diverse number of consultants who treated our patients, following a pattern of prescribing which favored treatment of symptoms, could also explain the differences in the prevalence of pDDIs in our sample compared to previous studies.

High numbers of comorbidities (hypertension [25], hyperlipidemia [26], diabetes mellitus [27], chronic kidney disease [28], arrhythmia [29], heart failure [30], epilepsy [31] significantly affected the frequency of pDDIs in previous studies. A high frequency of pDDIs was registered in patients with cardiac disorders, up to 91.6% [32] which was similar to our results where a large percentage of patients had cardiac comorbidities. The frequency of pDDIs in elderly patients who often have altered pharmacokinetics and pharmacodynamics, as well as impaired autoregulatory mechanisms, is significantly higher due to them being usually prescribed with multiple medications. Thus, they are at particularly increased risk for the occurrence of ADRs [32]. In our sample majority of patients were aged ≥65 years which contributed to the high frequency of pDDIs.

The average number of different drugs prescribed to our patients was significantly higher than in most other studies, although one study showed an average number of drugs of 17.09, which was similar to our study. In that study, pDDIs occurred in 90.02% of patients [10]. Goldberg et al showed that the risk of DDI in patients taking two different drugs was 13%, and this risk increased to 82% in patients taking 7 or more drugs concomitantly [33]. A sample of 8000 outpatients showed that the average number of drugs per patient had significantly increased in time, from 9.05 (1983-1984) to 14.6 (2003-2004) and consequently, the risk was higher for severe pDDIs [34]. A very large proportion of our patients had major polypharmacy imposing an increased risk of “prescribing cascade” (i.e. additional drug prescribing in order to treat adverse effects of drugs), whereby both phenomena could possibly clarify the high prevalence of pDDIs/pCDDIs in our study.

Most ICU patients receive antibiotic therapy similarly to our sample of patients (Table 1), and studies have shown a very high frequency of pDDIs which include antimicrobial therapy, of up to 46% [16]. The most common pCDDI, in our sample, was a combination of ceftriaxon and Ringer’s solution (that may cause intravascular precipitation of calcium-ceftriaxone complex) [35]. The most common pCDDI in the study of Teka et al was clarithromycin-simvastatin, which we recorded in 2.8% of our patients [36].

Rodriques et al showed that metoclopramide was the most common drug included in pCDDIs [18], while in our patients it appeared in 26.67% of different pCDDIs.

Although one study showed that NSAIDs do not affect the efficacy of SSRIs, and that this combination should not be avoided [37], a recent study reported an increased risk of intracranial hemorrhage in patients taking these groups of drugs concomitantly [38].

Similarly to our investigation, in a study by Uijtendaal et al, 20 pDDIs pairs were responsible for more than 90% of all pDDIs at admission to ICU [39]. Besides, ten pDDIs cover two-thirds of total exposure time to DDIs in another study [40]. This is one of the reasons that can help to reduce the number of pDDIs by better information and education of neurologist about drugs that are involved in the highest percentage of pDDIs [41].The most common major pDDIs, such as aspirin-diclofenac, diclofenac-enoxaparin, clopidogrel-diclofenac, may increase the risk of bleeding, and therefore it is necessary to monitor clinical features, laboratory parameters (PT, aPTT, INR) in these patients [38]. Simultaneous use of diclofenac-furosemide and aspirin-furosemide may be nephrotoxic and requires careful monitoring of renal function [4].

Frequency of pCDDIs in our patients was higher than the frequency of pCDDIs observed in prior studies (2.08%, in psychiatric patients [43] and 9.2% in patients hospitalized in internal medicine wards [44]). A similar frequency of pCDDIs of 13.4% was observed in patients with CKD [28].

In our sample there were 15 different pCDDIs, resembling the study by Rodriques et al conducted at the ICU, where 12 different pCDDI were observed [1]. ANCESTRAL-ED study, which was conducted on 3473 patients on admission to the emergency department (ED), showed 67 different pCDDIs, which is higher than in our sample, which is probably due to a larger number of patients included in the study, so less frequent pCDDIs have been discovered [45].In previous studies, the length of hospitalisation [1,6] was shown as a significant risk factor for pDDIs. In our results, the final model of logistic regression eliminated this factor, although the length of hospitalisation in NICU was statistically longer in patients with the occurrence of pCDDIs.

Furthermore, a high prevalence of pDDIs was previously demonstrated in patients with DM [27]. The incidence of disturbances of glucose level during stay in NICU ranged up to 40%, [46] as it was observed in our patients (45.9%). These patients received therapy for elevated glycemic values and therefore, this factor was found to be significantly associated with pCDDIs in univariate logistic regression analysis, but after adjustment for other independent and confounding variables, its importance disappeared.

Crude OR pointed to dual antiplatelet therapy as an important risk factor for pCDDIs, but in the multivariate analysis such a significance disappeared. Slightly more than one fifth of our patients had aspirin-clopidogrel pDDIs which is one of reasons of high frequency of pDDIs in stroke patients [8]. Meta-analyses showed that use of dual antiplatelet in acute ischemic stroke reduces the risk of recurrent stroke but long-term dual antiplatelet treatment increases the risk of systemic and intracranial hemorrhage [47]. However, recently published guidelines clearly recommended the regular use of combined aspirin and clopidogrel up to 10-21 days in patients with minor stroke to reduce the risk of recurrent stroke, but not in patients with major stroke due to a high risk of intracranial bleeding [48]. We believe that adherence to this recommendation by competent physicians may explain our finding, since the incidence of minor stroke in our environment correlates with the number of patients in our study in which such an interaction was observed.

In our study we identified two key factors contributing to the occurrence of pCDDIs: the number of prescribed drugs and the use of antipsychotics. The number of prescribed drugs was the most common factor associated with DDI in previous studies [1,2,6,8,10]. However, in the present study, we made one step forward in terms of confirming its influence on the occurrence of pCDDIs in patients with ischemic stroke. At that, as previously mentioned, the vast majority of our participants were prescribed with cardiovascular drugs, NSAIDs and antibiotics with a tendency to be involved in pCDDIs.

In patients with psychiatric comorbidity, prevalence of pCDDIs was 2.8% and that of pDDIs 81.65%, probably due to prescribing antipsychotic drugs that have great potential for interactions [43]. However, in our sample the prevalence of pCDDIs involving antipsychotics was significantly higher,. Risperidone, as the most frequently prescribed antipsychotic drug, has great potential for drug-drug interactions, especially with antidepressants, anticonvulsants, statins, azole antimycotic and antivirals [49]. On the other hand, there are data from observational studies reporting its significant influence on the incidence of stroke in older adults [50]. Eventually, a great caution and special monitoring of pCDDIs is advised in patients who are treated with a certain drug from the group of antipsychotics, such as risperidone.

## 5 Study limitations

The main limitation of the study lies in its retrospective design, so any inaccurate or incomplete information from the medical files could affect the observed outcomes and possibly lead to imprecise conclusions. Furthermore, this is a study on potential DDIs that have been detected based on theoretical knowledge, not based on actual clinical events. The research was conducted in one center, which potentially can influence the generalizability of the results. A significant percentage of medical files were excluded from the analysis in accordance with exclusion criteria which could influence the results of this research. Only one pDDI online checker (Micromedex^®^) was used, although certain differences were found between various sources of knowledge about pDDIs. However, we believe that the use of other online tools could not significantly contribute to the misclassification of patients with pDDIs/ pCDDI, as these tools were found to have reduced diagnostic performance compared to Micromedex^®^ [3]. In addition, like other online checkers, Micromedex^®^ does not take into account data such as dose of prescribed drug, frequency of drug administration, route of administration, duration of drug use, as well as clinical condition of patient and comorbidity. We did not have information about the number of different physicians who administered the therapy, which may be a risk factor for exposure to pDDIs, although all physicians always have an insight into a complete list of drugs that the patients received. PDDIs involving Alteplase^®^, as a first line pharmacotherapy for acute ischemic stroke, but which can substantially increase the risk of major bleeding when combined with antiplatelets and/or anticoagulants, were not observed in any our patient, because those who were treated with Alteplase^®^ did not receive any other medicine simultaneously that could interact with it for the next 24 hours. The study was conducted in neurological hospital settings and in patients with stroke, so the results could have limited generalizability to patients in other settings and with other diagnoses. Finally, there is also the issue of residual confounding, because there were some factors that we could not collect, like the severity of comorbidities the patients had, and patients with some rare forms of infarction were not taken into account in the first place.

## 6 Conclusions

The high frequency of pDDIs in patients with stroke can have a great clinical relevance, especially when a lot of patients are exposed to pCDDIs which could considerably contribute to serious adverse drug events. The number of prescribed drugs is a significant risk factor for exposure to the pCDDI, so each new drug must be added to therapy with special caution. Full attention should be paid when neurologists decide to prescribe antipsychotics, especially risperidone, in patients with stroke.
